# Association of serum CD14 level and functional polymorphism C-159T in the promoter region of CD14 gene with allergic rhinitis

**DOI:** 10.1007/s10238-023-01097-y

**Published:** 2023-06-07

**Authors:** Mai A. Kamel, Elham S. Selim, Enas A. Tantawy, Aya Elgendy, Alsayed Abdulmageed, Reham H. Anis

**Affiliations:** 1 Medical Microbiology and Immunology Department, Faculty of Medicine, Zagazig University, Zagazig, Egypt; 2 Internal Medicine/Allergy and Clinical Immunology Department, Faculty of Medicine, Ain Shams University, Cairo, 11591 Abbasia Egypt; 3 Department of Otorhinolaryngology, Faculty of Medicine, Zagazig University, Zagazig, Egypt

**Keywords:** Allergic rhinitis, Polymorphism, CD14, C-159T

## Abstract

Allergic rhinitis (AR) is an inflammatory disease of the upper respiratory tract affecting a significant number of the world’s population. It occurs as an IgE-mediated immune response of the nasal mucosa to inhaled allergens. The human Cluster of Differentiation 14 (CD14) is a glycosyl-phosphatidylinositol-anchored molecule expressed on the surface of monocytes and macrophages and functions as a receptor to lipopolysaccharides and inhaled endotoxins that may stimulate interleukins production by antigen-presenting cells. Consequently, CD14 plays a substantial role in allergic diseases and may become one of their etiological causes. This study aimed to determine the association between C-159T polymorphism in the CD14 gene promoter region and serum CD14 levels and the risk of Allergic rhinitis Egyptian patients and to test the validity of serum CD14 level measurement in predicting AR. This case–control study included 45 patients with AR referred to Allergy and Immunology Unit, Zagazig University Hospital, Zagazig, Egypt, and 45 healthy subjects as controls. Serum CD14 levels were measured by ELISA. The polymerase chain reaction-restriction fragment length polymorphism technique was used to detect C-159T gene polymorphism in the CD14 promoter region. There was a significant association between CD14 serum levels and AR incidence (*P* < 0.001), with patients having higher serum CD14 levels than controls. In addition, a significant association (*P* < 0.001) was detected between serum CD14 levels and the severity of AR, as well as elevated serum CD14 levels in severe and the most severe cases. On the molecular level, there was a statistically significant relationship between patients and the control group regarding the CD14 genotype (*P* < 0.001), where CT and TT genotypes and T allele were primarily associated with the cases group, indicating that the risk of AR was significantly associated with the inheritance of the TT genotype. Additionally, a statistically significant association was found between the severity of AR and CD14 genotype (*P* < 0.001), where TT genotypes were mainly associated with severe and the most severe cases. In the studied groups, there was a statistically significant difference (*P* < 0.05) between the CD14 genotype and serum CD14 levels, with TT genotypes being associated with higher CD14 levels. The results obtained in this study revealed that serum CD14 level is a potential biomarker for the diagnosis of AR and, at the genetic level, a potential predictor of disease.

## Introduction

Allergic rhinitis (AR) is an inflammatory disease of the upper respiratory tract characterized by nasal congestion, watery nasal discharge, itchy nose, and sneezing. It is caused by IgE-mediated reactions against inhaled allergens [1, 2]*.* Epidemiological studies have reported that nearly 10–40% of the global population is affected by AR, with increasing incidence [3]. The etiology of AR remains undetermined [4]. However, the disease is associated with multiple genetic and environmental factors [5].

Several studies uncovered a complex network of soluble and membrane-bound factors that regulate allergic responses. One of these factors is Cluster of Differentiation 14 (CD14), a glycosyl-phosphatidylinositol-anchored peptide generated primarily on the surfaces of monocytes and macrophages [6, 7]. It induces antigen-presenting cells to produce interleukins by acting as a receptor for lipopolysaccharides and inhaled endotoxins [8]. It also serves as a signaling molecule for the human eosinophil interleukin 5 (IL-5), interleukin 3 (IL-3), and granulocyte-monocyte colony-stimulating factor (GM-CSF) receptor subunits [9].

A genome-wide search for vulnerable loci modifying allergic reactions has identified CD14 as a significant candidate gene in allergic reactions [10]. A C-to-T transition was identified in the promoter region of this gene at location 159. This vital polymorphism C-159T was associated with a variety of allergic diseases [11]. In this context, to our knowledge, this is the first study to investigate the association between serum level of sCD14 and CD14 gene polymorphism C-159T as well as AR disease in a group of the Egyptian population.

## Methodology

### Study design and setting

A case–control study was carried out over a one-year period from June 2020 to June 2021 in the Department of Medical Microbiology and Immunology, Faculty of Medicine and Allergy & Immunology Unit, Zagazig University hospitals, Egypt. The current study included 45 patients with AR referred to the Allergy and Immunology Unit and 45 controls. The sample size was calculated using the online tool Open epi version 3.1 [12].

This study was approved by the Institutional Review Board (IRB) of Zagazig University, Faculty of Medicine (IRB reference number: ZUIRB# 6083/5-2020). It was carried out according to Helsinki Declaration guidelines. Informed consent was obtained from all study participants.

### Study subjects

This study included 90 subjects (45 in the case group and 45 in the control group). The mean age was 32.62 ± 9.96 in the case group and 32.09 ± 9.39 in the control group. They were enrolled from the Allergy and Immunology Unit, Department of Medical Microbiology and Immunology, Faculty of Medicine, Zagazig University, Egypt. Inclusion criteria: adult patients > 18 years old with typical nasal symptoms and positive skin prick test, in addition to patient consent to participate in the study. Exclusion criteria included patients diagnosed with AR associated with rhino sinusitis, asthma, or systemic diseases and patients who used systemic corticosteroids or antihistamines within the last month. The control group included apparently healthy individuals with no history of allergic diseases.

The allergens exposure history confirmed diagnosis of allergy, allergic diseases running in the family, and accurate clinical examination for typical nasal symptoms, which included nasal congestion, watery nasal discharge, nasal itching, sneezing, and postnasal drip.

Rhinitis was classified according to the Japanese guidelines for AR 2016 (Revised 8th Edition) [13]. On the basis of the severity of nasal obstruction, sneezing, and rhinorrhea, cases of AR were categorized as the most severe, severe, moderate, and mild.

### Skin test

Skin prick test was performed at the volar aspect of the forearm, one drop of each allergen extract from a panel containing: house dust mite, tobacco leaf, wool, maize pollens, cotton, mixed molds, grass pollens, date palm pollens and hay dust (Allergy Laboratories Inc., Oklahoma City, USA) was applied to the skin. The skin test was done at least 3 cm apart, according to a previously established protocol [14, 15]. Histamine and saline were used as the positive and negative controls, respectively. The skin test result was determined by measuring the diameter of the wheal formed after 20 min. A wheal with a diameter of 3 mm or more accompanied by erythema was considered positive.

### Specimen collection

Five milliliters of venous blood sample was taken from each participant. Three milliliters was collected in a plain tube to separate the serum for measuring the serum level of sCD14. The remaining 2 ml of the blood sample was collected in Ethylene-diamine-tetra acetic acid (EDTA) containing tube and utilized for DNA extraction and identification of CD14 polymorphism C–159T [8, 16].

### Determination of serum levels of sCD14

Serum levels of sCD14 were measured using a commercially available sandwich enzyme-linked immunosorbent assay (ELISA) kit (ELISA KIT, SunRed, Shanghai, China) in accordance with the manufacturer’s instructions.

### Identification of CD14 polymorphism C–159T

**Fig. 1:**
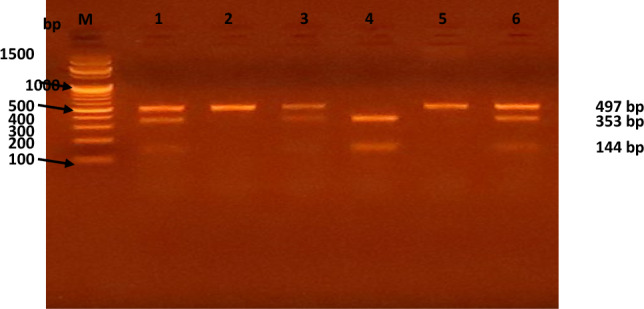
Image of Gel electrophoresis shows FastDigest Eco47I restriction patterns of CD14 gene of six cases. M: DNA ladder (100–1500 bp), Lanes 2 and 5: Homozygous wild [CC] type indicated by a single 497 bp band; lanes 1, 3 and 6: Heterozygous mutant [CT] type indicated by 144 bp, 353 bp and 497 bp bands; lane 4: Homozygous mutant [TT] type indicted by 144 bp and 353 bp bands

C-159T distribution was determined using polymerase chain reaction-restriction fragment length polymorphism (PCR-RFLP) analysis. A commercially available DNA extraction kit (Thermo Fisher Scientific, Waltham, Massachusetts, USA) was used to extract genomic DNA from blood collected in EDTA-containing tubes. Extraction of genomic DNA from blood collected in EDTA-containing tubes was carried out using a commercially available DNA extraction kit (Thermo Fisher Scientific, Waltham, Massachusetts, USA). Using a modified form of the procedure that Baldini et al. previously published, C-159T genotyping was discovered [17]. CD14 forward primer (5′-GTG CCA ACA GAT GAG GTT CAC-3′) and CD14 reverse primer (5′-GCC TCT GAC AGT TTA TGT AAT-3′) provided by (Thermo Fisher Scientific, Waltham, Massachusetts, USA) were used underneath the subsequent response situations: preliminary denaturation (for 5 min at 96 °C), then 36 cycles of 95 °C (30 sec), 68 °C (30 sec), 72 °C (30 sec), and finally a final extension phase of 72 °C for seven minutes. Additionally, 1.5 μl of FastDigest Eco47I (Thermo Fisher Scientific, Waltham, Massachusetts, USA) was used to digest 3 μl of the 497-bp PCR product overnight at 37 °C. The FastDigest Eco47I endonuclease can precisely recognize the GGTCC sequence to cut the 497 bp product into two fragments of 144 bp and 353 bp. Only those with the CD14/-159T allele are eligible for recognition at this site. Therefore, the homozygous CC genotype is identified by a single 497 bp band, and the homozygous genotype is characterized by two bands of 144 bp and 353 bp, while the heterozygous CT genotype displays all three bands, as demonstrated in Fig. 1. The processed fragments were resolved using a 2.5% agarose gel and visualized with ethidium bromide.

### Statistical analysis

All data were collected, tabulated, and statistically analyzed using SPSS 26.0 for Windows (SPSS Inc., Chicago, IL, USA). Quantitative data were expressed as median (interquartile range), and qualitative data were expressed as absolute frequencies (number) and relative frequencies (percentage). Mann–Whitney U test was used to compare between two groups of non-normally distributed variables. Kruskall–Wallis test was used to compare more than two independent groups of non-normally distributed variables. Categorical variables were compared using the Chi-square test (*X*2) for testing genotypes and alleles frequencies. Roc curve was done to determine sensitivity and specificity. All tests were two sided. *p*-value < 0.05 was considered statistically significant (S), *p*-value ≥ 0.05 was considered statistically insignificant (NS).

## Results

### Study population

The case group in the current study included 22 male patients and 23 female patients with AR, with a mean age of 32.62 ± 9.96 years. The control group included only healthy volunteers, 23 males and 22 females, with a mean age of 32.09 ± 9.39 years. No significant difference was observed between the studied groups regarding age (*P* = 0.794) and sex (*P* = 1.0).

### Symptoms and skin prick test results

The results are presented in Table 1. In terms of AR symptoms, most patients presented with nasal discharge (93.3%). Other symptoms included sneezing (86.7%), nasal obstruction (75.6%), and itching (60%), with postnasal drip (11.1%) being the least common.

**Table 1:** Frequency distribution of symptoms and positive skin test to tested allergens among the case group (* n*  =  45)

Symptoms	Case group (* n* = 45)		Positive skin test to tested allergens	Case group(* n* = 45)	
	*n*	%		*n*	%
Discharge	42	93.3	House dust mite	35	77.8
Itching	27	60	Tobacco leaf	14	31.1
Obstruction	34	75.6	Wool	4	8.9
Sneezing	39	86.7	Maize pollens	34	75.6
Postnasal drip	5	11.1	Cotton	4	8.9
			Mixed molds	7	15.6
			Grass pollens	31	68.9
			Date palm pollens	34	75.6
			Hay dust	9	20

The outcomes of the skin prick test revealed that the majority of patients were allergic to house dust mites (77.8%), followed by maize pollens and date palm pollens with equal percentages (75.6%), while the minimum frequent allergens among patients in this study were wool and cotton with equal percentage (8.9%), as depicted in Table 1.

### Serum level of sCD14 and CD14 genotyping

**Fig. 2:**
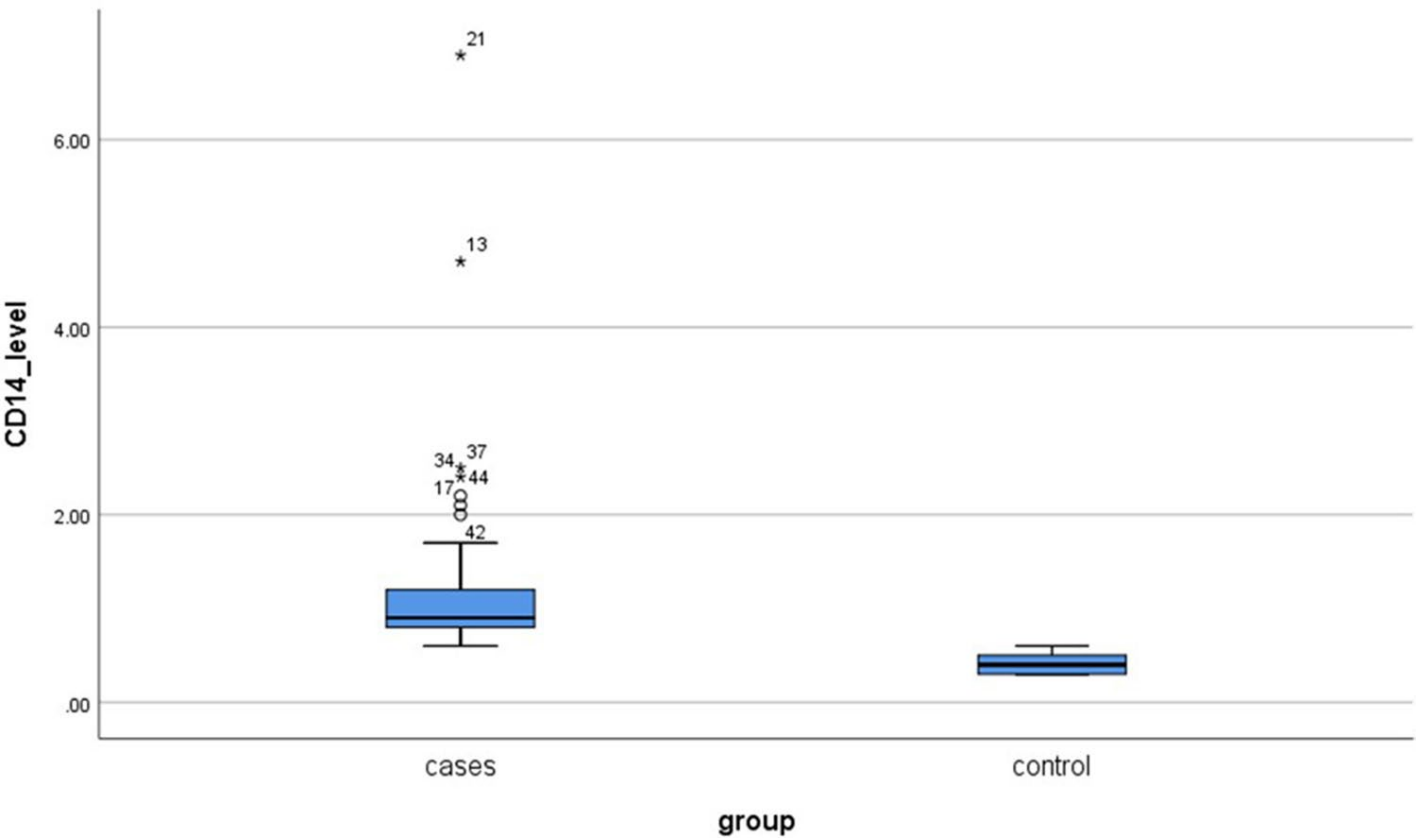
Box blot illustrating the median values and interquartile distances of serum levels of CD14 (ng/ml) in the case and control groups

As demonstrated in Fig. 2, the median (IQR) sCD14 serum level was significantly higher in AR patients 0.9(0.8–1.3) ng/ml than in controls at 0.4(0.3–0.5) ng/ml (*P* < 0.001).

In addition, a statistically significant difference (*P* < 0.001) was found in the severity of AR and serum sCD14 levels among cases with higher serum sCD14 levels in severe and most severe cases, as shown in Table 5.

Regarding possible risk factors of AR, there was a statistically significant difference in the serum sCD14 levels in the cases regarding sex, residence, and smoking habit, with higher serum sCD14 levels in males, rural residents, and smokers (*P* < 0.05), as shown in Table 2.

**Table 2:** Relation between possible risk factors of AR and serum sCD14 level among cases

Items		Serum sCD14 level	Mann–Whitney U Test	*P* value
		Median (IQR)		
Sex	Male (* n* = 22)	1.3 (0.8–2.13)	− 3.4	0.001*
	Female (* n* = 23)	0.8 (0.7–0.9)		
Residence	Rural (* n* = 25)	1.1(0.9–2.05)	− 4.8	< 0.001*
	Urban (* n* = 20)	0.8 (0.7–0.8)		
Smoking	Yes (* n* = 13)	2 (1.5–2.45)	− 5.2	< 0.001*
	No (* n* = 32)	0.8 (0.7–0.9)		

The test used is Mann–Whitney U test

* the p-value is statistically significant

**Fig. 3:**
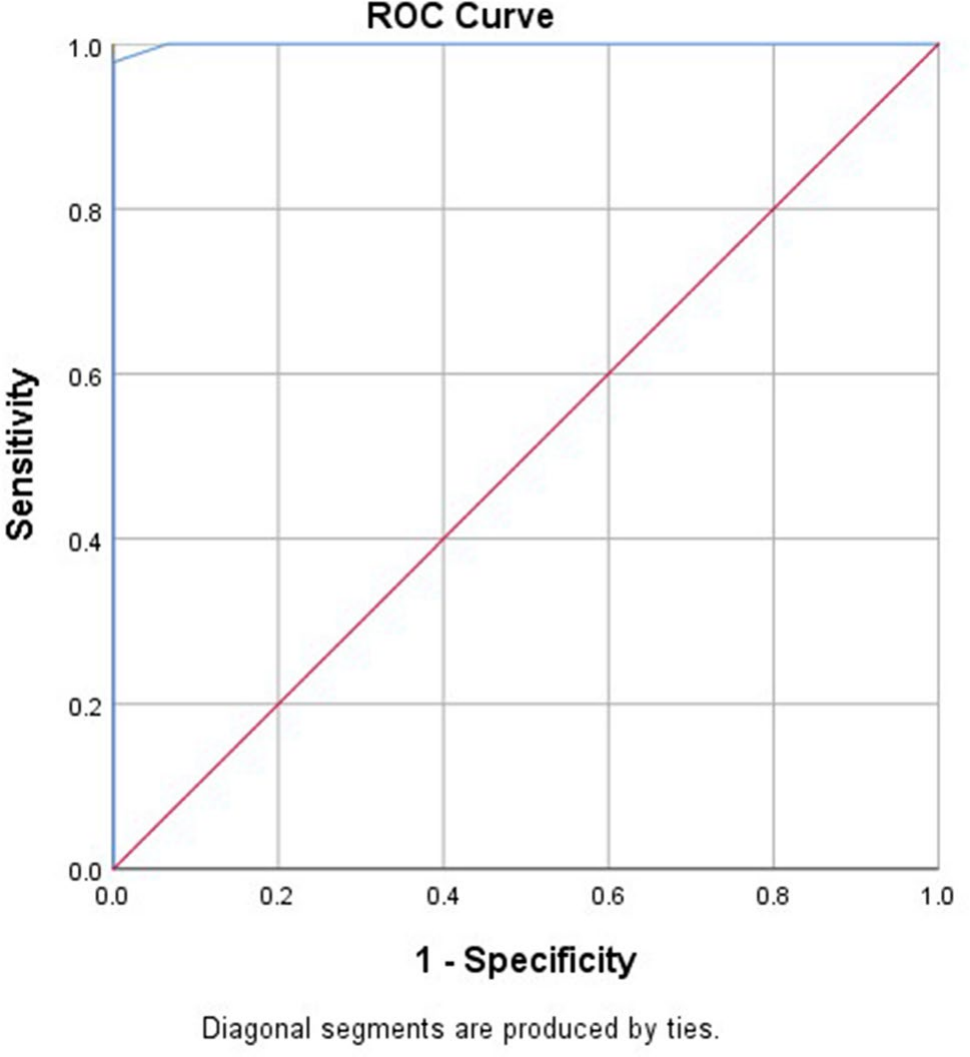
Roc curve illustrating the validity of serum sCD14 level in predicting AR

By testing the validity of serum sCD14 level measurement in predicting AR, the value of sensitivity at a cutoff of 0.65 was (97.8%), specificity = (100%), predictive value for positive (PVP) = (100%), predictive value for negative (PVN) = (97.8%), and (98.9%) accuracy, as depicted in Fig. 3.

In the control group, the genotypic prevalence of the CD14 polymorphism C-159T was 40% (* n* = 18) for CC, 53.3% (* n* = 24) for CT, and 6.7% (* n* = 3) for TT genotype. Among AR patients, CC was 4.4% (* n* = 2), CT was 62.2% (* n* = 28), and TT genotype was 33.3% (* n* = 15), as illustrated in Table 3. Concerning CD14 genotype, it showed a statistically significant association between AR patients and the control group (*P* < 0.001), where CT and TT genotypes and T allele were mainly associated with the case group showing a significant association between that the risk of AR and the inheritance of TT genotype (*P* < 0.001, OR (CI) 3.6 (1.95–6.7).

**Table 3:** Comparing CD14 genotypes between the studied groups (* n* = 90)

Variables	Case group (* n* = 45)	Control group (* n* = 45)	*P* value	Sig	OR (CI)
Genotypes					
CC	2 (4.4%)	18 (40%)	< 0.001*	Ref	
CT	28 (62.2%)	24 (53.3%)		0.001*	10.5 (2.208–49.93)
TT	15 (33.3%)	3 (6.7%)		< 0.001*	45 (6.624–305.69)

Alleles	*n* (%)	*n* (%)	*P*-value	OR (CI)
C	32 (35.6)	60 (66.7)	< 0.001*	3.6 (1.95–6.7)
T	58 (64.4)	30 (33.3)		

The test applied is the Chi-Square test

* the p-value is statistically significant

No statistically significant difference was detected between CD14 genotype and either different symptoms or positive skin test for different tested allergens (*P* > 0.05), as depicted in Table 4.

**Table 4:** Relation between symptoms of AR and positive skin test to tested allergens among different CD14 genotypes of the studied groups

Variables		Category	CC (* n* = 2)		CT (* n* = 28)		TT (* n* = 15)		Chi-Square test	*P* value
			*n*	%	*n*	%	*n*	%		
Symptoms of AR	Discharge	No	0	0	3	10.7	0	0	1.95	0.377
		Yes	2	100	25	89.3	15	100		
	Itching	No	1	50	8	28.6	9	60	4.10	0.128
		Yes	1	50	20	71.4	6	40		
	Obstruction	No	0	0	8	28.6	3	20	1.06	0.587
		Yes	2	100	20	71.4	12	80		
	Sneezing	No	1	50	5	17.9	0	0	5.13	0.077
		Yes	1	50	23	82.1	15	100		
	Postnasal drip	No	1	50	24	85.7	15	100	5.22	0.073
		Yes	1	50	4	14.3	0	0		
Allergenicity to tested allergens	Dust	No	0	0	5	17.9	5	33.3	1.95	0.377
		Yes	2	100	23	82.1	10	66.7		
	Smoke	No	1	50	21	75	9	60	1.37	0.503
		Yes	1	50	7	25	6	40		
	Wool	No	1	50	25	89.3	15	100	5.75	0.056
		Yes	1	50	3	10.7	0	0		
	Maize pollens	No	1	50	6	21.4	4	26.7	0.88	0.642
		Yes	1	50	22	78.6	11	73.3		
	Cotton	No	2	100	25	89.3	14	93.3	0.40	0.818
		Yes	0	0	3	10.7	1	6.7		
	Mixed molds	No	2	100	23	82.1	13	86.7	0.53	0.764
		Yes	0	0	5	17.9	2	13.3		
	Grass pollens	No	0	0	7	25	7	46.7	3.08	0.214
		Yes	2	100	21	75	8	53.3		
	Palm pollens	No	1	50	9	32.1	1	6.7	4.17	0.124
		Yes	1	50	19	67.9	14	93.3		
	Hay dust	No	2	100	21	75	13	86.7	1.35	0.508
		Yes	0	0	7	25	2	13.3		

The test applied is the Chi-Square test

Table 5 depicts a statistically significant association between the CD14 genotype and AR severity (*P* < 0.001). TT genotypes were associated with severe and most severe cases, CT was associated with mild and moderate cases, and CC was associated only with mild cases. Furthermore, a statistically significant difference (*P* < 0.001) was detected between the CD14 genotype and serum sCD14 levels of the studied groups, where TT genotypes were associated with higher serum sCD14 levels.

**Table 5:** Relation between severity of AR and serum sCD14 levels among different genotypes of the studied groups

Severity of AR		Mild (*n* = 22)		Moderate (*n* = 8)		Severe (*n* = 8)		Most severe (*n* = 7)		Chi-Square test	*P*-value
		*n*	(%)	*n*	(%)	*n*	(%)	*n*	(%)		
Genotypes	CC	2	9.1	0	0	0	0	0	0	46.16	< 0.001*
	CT	20	90.9	8	100	0	0	0	0		
	TT	0	0	0	0	8	100	7	100		

		Serum sCD14 Level	Kruskal–Wallis test	*P*-value
		Median (IQR)		
Genotypes	CC	0.65 (0.6–0.65)	32.3	< 0.001*
	CT	0.8 (0.73–0.9)		
	TT	1.7 (1.2–2.4)		

The tests applied are the Chi-Square test and the Kruskal–Wallis test

* the p-value is statistically significant

## Discussion

AR is a worldwide health problem with unknown etiology [18], but it has a strong association with genetic and environmental factors in etiology [5, 19].

In the current study, a skin prick test was performed on Egyptian patients with AR. It demonstrated that most cases were allergic to house dust mites (77.8%), followed by maize pollens and date palm pollens equally (75.6%). In their study, Marzouni et al. reported that the most frequent allergens among Iranian patients with AR were lamb’s quarters (82.4%) and Salsola kali (90.4%). The findings of previous research studies are controversial, which may be due to different geographical distribution and different environmental conditions [8].

Several studies were conducted to determine the factors involved in the pathogenesis of AR. One of these factors was CD14 which acts as a receptor for lipopolysaccharide and inhaled endotoxin. It has a significant role in immune responses in different allergic diseases [10, 20]. On the genetic level, a polymorphism was recognized inside the CD14 gene promoter sequence with a C–T transition at base pair-159 from the main transcription start site [21]. The wild type was (CC), while the homozygous mutant was (TT), and the heterozygous mutant was (CT) [22]. Several studies reported the association between this polymorphism and allergic diseases [23, 24].

To our knowledge, this study is the first to investigate the association between serum sCD14 levels and the C-159T functional polymorphism of the CD14 gene in relation to the prevalence and severity of AR in Egyptian patients.

Regarding AR symptoms, this study showed that the most prevalent symptom among AR patients was nasal discharge (93.3%). These results agree with those reported by Marzouni et al. [8] who found that nasal discharge was the most common symptom of AR (91.2%).

In agreement with Marzouni et al., our study also revealed that serum sCD14 levels were significantly higher in AR patients than in controls (*P* < 0.001) [9]. On the contrary, several investigations found that atopic patients’ serum sCD14 levels were lower than those of non-atopic individuals [10]. Additionally, numerous authors reported no difference in serum sCD14 levels comparing atopic individuals and those who were not [25]. Discrepancies in these results may be due to methodological differences between studies, different populations, or different phenotypes of allergy.

Furthermore, our study reported a statistically significant difference (*P* < 0.001) between the severity of AR and serum sCD14 levels among cases with higher serum sCD14 levels in severe and most severe cases. A previous study on allergic asthma patients indicated that serum sCD14 levels were negatively associated with asthma severity [20]. These contradicting findings are more likely due to differences between the pathophysiology of asthma and AR, as well as the impact of microorganisms and allergen exposure associated with CD14 and CD14 gene polymorphisms.

By examining the effects of various risk factors for AR on serum sCD14 levels, our study revealed higher serum sCD14 levels in male patients compared with female patients. This result is consistent with the findings of Prester et al. and Levan et al. [10, 26], which suggest that atopy disturbs innate immunity differently in men and women, suppressing activity in CD14 more in women than in men.

Regarding the residence, our results showed that serum sCD14 levels were higher in patients raised in rural areas than those raised in urban areas, which agrees with earlier similar results reported by other studies. This finding suggests that CD14 could act as a biological marker of exposure to greater bacterial loads, which may clarify the higher sCD14 serum levels in patients raised in rural places [27].

People are exposed to severe health risks, such as air pollutants from tobacco smoke. A number of studies indicated that active and passive smoking might increase the risk of atopic disease [28]. By examining the effect of smoking habit on serum sCD14 levels in cases, our study found that serum sCD14 levels were higher in smokers than nonsmokers. A previous study reported similar results [29]. This finding can be elucidated by a sustained introduction to bacterial endotoxins in tobacco smoke and the interface of genes and smoking [30, 31]. Additionally, Prester et al. discovered no relationship between CD14 serum levels and active smoking behavior. This result can be attributed to the younger age and shorter smoking history of the patients in their study [10].

At the molecular level, we investigated the connection between the functional polymorphism C-159T in the CD14 gene’s promoter region and the prevalence of AR. We found a statistically significant relationship between AR patients and controls with respect to the CD14 genotype.

In the current study, CT and TT genotypes were predominantly associated with case groups compared with CC genotypes, indicating that AR risk is significantly associated with carriers of the T allele. This finding is consistent with a study by Marzouni et al. [8]. However, other studies have reported a different finding of a lower incidence of AR in patients with CT and TT alleles [24]. Yazdani et al. revealed that the CC genotype is mainly related to allergic asthma risk 17. According to a study conducted on a Ukrainian population, the carriers of the C allele at the polymorphic region C-159T of the CD14 gene were at increased risk of atopic dermatitis compared with carriers of the T allele [11]. The study also revealed an association between the CD14 gene polymorphism C-159T and atopic disease [24, 32].

Our study did not find a significant relationship between AR symptoms and CD14 genotypes (*P* > 0.05). Furthermore, no statistically significant differences (*P* > 0.05) were found between different CD14 genotypes and different types of allergens tested. In contrast, previous studies on the Iranian population reported an association between susceptibility to several tested allergens in AR patients and the CD14 polymorphism C-159T. This finding indicates that patients with the CT and TT genotypes are more susceptible to salsola kali, ash, and millet allergens and display significantly more admixture than the CC genotype patients [8].

In our study, there was a significant association between the severity of AR and CD14 genotype (*P* < 0.001), where TT genotypes were associated with severe and most severe cases. In contrast, CT was associated with mild and moderate cases, and CC was associated only with mild cases. Furthermore, a significant association (*P* < 0.001) between the CD14 genotype and serum sCD14 levels of the studied groups was found with the TT genotypes associated with higher serum sCD14 levels. In agreement with our results, de Faria et al. showed a significant association between the TT genotype of CD14 (− 159 C/T) polymorphism and severe asthma [33]. However, Nieto-Fontarigo et al. [6] reported an association between the T allele and TT genotype of CD14 (− 159 C/T) polymorphism with reduced risk of moderate-severe allergic asthma. According to Ghosh et al. [10] there is a substantial association between the severity of asthma and the distribution of CD14 genotypes. Consistent with our results, previous studies revealed that people with the TT genotype had significantly higher serum levels of sCD14 than people with the CC and CT genotypes [6, 34].

These contradictory results usually characterize studies concerning the genetics of allergic diseases. These conflicting results can be attributed to a lack of statistical power, differences in ethnicity and age, genetic heterogeneity in different populations, and varying environmental conditions. Multiple gene–gene interactions, as well as differences in the pathophysiology of AR and other allergic phenotypes, may also contribute to these differences.

## Conclusion

In conclusion, this study demonstrated that serum sCD14 could be a potential biomarker in the pathogenesis of AR. It is possible that type C-159T influences the incidence and severity of AR, could aid in disease prediction, and represent a new treatment option.

## Limitations of the study

The fact that this study’s findings are based on data from only a small number of patients is a significant limitation. Therefore, additional research is required, including larger sample size.

## References

[1] Hao Y, Wang B, Zhao J (2022). Identification of gene biomarkers with expression profiles in patients with allergic rhinitis. Allergy Asthma Clin Immunol.

[2] Dahanayake JM, Perera PK, Galappaththy P, Samaranayake D (2020). Efficacy and safety of two Ayurvedic dosage forms for allergic rhinitis: study protocol for an open-label randomized controlled trial. Trials.

[3] Ding X, Huang S, Tang Y, Lin J (2021). Effectiveness and safety of ear acupuncture for allergic rhinitis: a protocol of randomized controlled trial. Medicine.

[4] Kortekaas Krohn I, Shikhagaie MM, Golebski K (2018). Emerging roles of innate lymphoid cells in inflammatory diseases: clinical implications. Allergy.

[5] Khan SJ, Dharmage SC, Matheson MC, Gurrin LC (2018). Is the atopic march related to confounding by genetics and early-life environment? A systematic review of sibship and twin data. Allergy.

[6] Nieto-Fontarigo JJ, Salgado FJ, San-Jose ME (2018). The CD14 (− 159 C/T) SNP is associated with sCD14 levels and allergic asthma, but not with CD14 expression on monocytes. Sci Rep.

[7] Ayaslioglu E, Kalpaklioglu F, Kavut AB, Erturk A, Capan N, Birben E (2013). The role of CD14 gene promoter polymorphism in tuberculosis susceptibility. J Microbiol Immunol Infect.

[8] Marzouni HZ, Farid-Hosseini R, Jabari-Azad F (2019). CD14 as a serum immune biomarker and genetic predisposition factor for allergic rhinitis. Iran J Otorhinolaryngo.

[9] Hamajima Y, Fujieda S, Sunaga H (2007). Expression of Syk is associated with nasal polyp in patients with allergic rhinitis. Auris Nasus Larynx.

[10] Ghosh A, Dutta S, Podder S (2018). Sensitivity to house dust mites allergens with atopic asthma and its relationship with CD14 C (-159T) polymorphism in patients of West Bengal, India. J Med Entomol.

[11] Litus O, Derkach N, Litus V, Bisyuk Y, DuBuske L (2018). The C-159T Polymorphism of the CD-14 Gene and cytokine profiles in adults with atopic dermatitis. Ann Allergy Asthma Immunol.

[12] Dean AG. OpenEpi: open source epidemiologic statistics for public health. http://www.OpenEpi*. com.* 2007.

[13] Okubo K, Kurono Y, Ichimura K (2017). Japanese guidelines for allergic rhinitis 2017. Allergol Int.

[14] Heinzerling L, Mari A, Bergmann K-C (2013). The skin prick test—European standards. Clin Transl Allergy.

[15] Tourlas K, Burman D (2016). Allergy testing. Prim Care Clin Off Pract.

[16] Yazdani N, Amoli M, Naraghi M (2012). Association between the functional polymorphism C-159T in the CD14 promoter gene and nasal polyposis: potential role in asthma. J Investig Allergol Clin Immunol.

[17] Baldini M, Carla Lohman I, Halonen M, Erickson RP, Holt PG, Martinez FD (1999). A Polymorphism* in the 5′ flanking region of the CD14 gene is associated with circulating soluble CD14 levels and with total serum immunoglobulin E. Am J Respir Cell Mol Biol.

[18] Canonica GW, Mullol J, Pradalier A, Didier A (2008). Patient perceptions of allergic rhinitis and quality of life. World Allergy Organ J.

[19] Samitas K, Carter A, Kariyawasam HH, Xanthou G (2018). Upper and lower airway remodelling mechanisms in asthma, allergic rhinitis and chronic rhinosinusitis: the one airway concept revisited. Allergy.

[20] Zhou T, Huang X, Ma J (2019). Association of plasma soluble CD14 level with asthma severity in adults: a case control study in China. Respir Res.

[21] Bashir SB, Dar AQ, Rasool R, Shamim R, Pandit A (2016). Impact of promoter CD14 C> T 159 gene single nucleotide polymorphism and outcome of sepsis. Bangladesh J Med Sci.

[22] Panda AK, Tripathy R, Das BK (2021). CD14 (C-159T) polymorphism is associated with increased susceptibility to SLE, and plasma levels of soluble CD14 is a novel biomarker of disease activity: a hospital-based case-control study. Lupus.

[23] Baranovskyi TP (2019). Association of the CD14 (-159C/T) SNP with house dust mites sensitization in patients with perennial allergic rhinitis in the population of ukraine. Allergy.

[24] Bisyuk Y, Dubovyi A, DuBuske I, Litus V, DuBuske LM. Association of the CD14 C159T and the toll-like receptor 4 Asp299Gly polymorphisms with various phenotypes of asthma in adults from Crimea. In: Allergy & asthma proceedings. 2020; 41(2).‏ 10.2500/aap.2019.40.190007 31530336

[25] Stockton ML (2003). High circulating soluble CD14 (sCD14) levels are not associated with asthma or atopy in an afro-Caribbean population from Barbados. J Allergy Clin Immunol.

[26] LeVan TD, Michel O, Dentener M (2008). Association between CD14 polymorphisms and serum soluble CD14 levels: effect of atopy and endotoxin inhalation. J Allergy Clin Immunol.

[27] Lauener RP, Birchler T, Adamski J (2002). Expression of CD14 and Toll-like receptor 2 in farmers' and nonfarmers' children. Lancet.

[28] Ciaccio CE, Gentile D (2013). Effects of tobacco smoke exposure in childhood on atopic diseases. Curr Allergy Asthma Rep.

[29] Reiner AP, Lange EM, Jenny NS (2013). Soluble CD14: Genomewide association analysis and relationship to cardiovascular risk and mortality in older adults. Arterioscler Thromb Vasc Biol.

[30] Rava M, Smit LA, Nadif R (2015). Gene–environment interactions in the study of asthma in the postgenomewide association studies era. Curr Opin Allergy Clin Immunol.

[31] Hussein YM, Shalaby SM, Zidan HE, Sabbah NA, Karam NA, Alzahrani SS (2013). CD14 tobacco gene–environment interaction in atopic children. Cell Immunol.

[32] Dębińska A, Danielewicz H, Drabik-Chamerska A, Kalita D, Boznański A (2019). Genetic polymorphisms in pattern recognition receptors are associated with allergic diseases through gene–gene interactions. Adv Clin Exp Med.

[33] De Faria IC, Faria EJD, Toro AA, Ribeiro JD, Bertuzzo CS (2008). Association of TGF-beta1, CD14, IL-4, IL-4R and ADAM33 gene polymorphisms with asthma severity in children and adolescents. Jornal de pediatria.

[34] Han D, She W, Zhang L (2010). Association of the CD14 gene polymorphism C-159T with allergic rhinitis. Am J Rhinol Allergy.

